# DEPRESSION AND PAIN PERSEVERANCE THROUGH EMPOWERED RECOVERY (DAPPER) PILOT PROGRAM: A CASE STUDY ON TECHNOLOGY

**DOI:** 10.1093/geroni/igac059.827

**Published:** 2022-12-20

**Authors:** Catherine Clair, Tonisha Melvin, Sarah Szanton, Martha Abshire Saylor, Roland J Thorpe, Jr., Janiece Taylor

**Affiliations:** Johns Hopkins Bloomberg School of Public Health, Baltimore, Maryland, United States; Johns Hopkins School of Nursing, Baltimore, Maryland, United States; Johns Hopkins University School of Nursing, Baltimore, Maryland, United States; Johns Hopkins University School of Nursing, Baltimore, Maryland, United States; Johns Hopkins University, Baltimore, Maryland, United States; Johns Hopkins University, Baltimore, Maryland, United States

## Abstract

There is a paucity of work focusing on the engagement of older African American women with technology. The aim of the pilot study was to test the Depression and Pain Perseverance through Empowered Recovery (DAPPER) program in a sample of older African American women living with pain and low mood (N=19). The research team hypothesized that most of the women would prefer virtual visits, thus both in-person and virtual options were available for program delivery. So far, of 11 participants, five women have opted to do in-person visits. When asked why, one participant said that having the nurse come to her house was “rewarding, [especially] when you live alone.” Another participant commented that she did not have a computer in her home. These findings demonstrate that older adults exist on a spectrum of comfort with and access to technology.

